# A biomechanical comparison of three fixation methods for unstable femoral neck fractures with medial calcar defect

**DOI:** 10.1186/s13018-023-04100-0

**Published:** 2023-08-22

**Authors:** Emre Koraman, Yusuf Iyetin, Oguzhan Ozyaman, Muhlik Akyurek

**Affiliations:** 1 Department of Orthopaedics and Traumatology, Faculty of Medicine, Demiroglu Bilim University Kadikoy Florence Nightingale Hospital, Feneryolu Mah, Bagdat Cad. No: 63, Kiziltoprak/Kadikoy/Istanbul, Turkey; 2 Department of Orthopaedics and Traumatology, Pendik Bolge Hospital, Istanbul, Turkey; 3 Department of Orthopaedics and Traumatology, Faculty of Medicine, Istanbul Medeniyet University Goztepe Prof. Dr Suleyman Yalcin City Hospital, Istanbul, Turkey; 4 Department of Orthopaedics and Traumatology, Maria-Josef Hospital, Greven, Germany

**Keywords:** Unstable femoral neck fractures, Medial calcar defect, Fixation techniques, Medial buttress plate, Biomechanical stability

## Abstract

**Background:**

Unstable femoral neck fractures with medial calcar defects are difficult to manage. The optimal fixation methods for these fractures have been a subject of ongoing debate among orthopedic surgeons. In this study, three different fixation techniques for vertical, medial defected femoral neck fractures were compared.

**Methods:**

In this study, a biomechanical analysis was conducted to compare three fixation methods: cannulated screws (Group 1), cannulated screws combined with a medial buttress plate (Group 2), and intramedullary nails (Group 3). Synthetic composite bone models representing vertical collum femoris fractures with medial calcar defects were used. Each group consisted of seven specimens, and, to maintain consistency, a single surgeon performed the surgical procedure. Biomechanical testing involved subjecting the specimens to axial loading until failure, and the load to failure, stiffness, and displacement values were recorded. Normality was tested using the Shapiro–Wilk test. One-way ANOVA and Tukey’s HSD post hoc test were used for comparisons.

**Results:**

The difference in the load to failure values was statistically significant among the groups, with Group 2 exhibiting the highest load to failure value, followed by Group 3 and Group 1. Stiffness values were significantly higher in Group 2 than in the other groups. Displacement values were not significantly different between the groups. Fracture and displacement patterns at the point of failure varied across the groups.

**Conclusion:**

The results of this study indicate that fixation with a medial buttress plate in combination with cannulated screws provides additional biomechanical stability for vertical femoral neck fractures with medial calcar defects. Intramedullary nail fixation also demonstrated durable stability in these fractures. These findings can be used to better understand current management strategies for these challenging fractures to promote the identification of better evidence-based recommendations.

## Background

Unstable, vertical fractures occurring in the femoral neck, particularly classified as Pauwels type 3 fractures, are frequently observed among young adults as a result of high-energy trauma [1]. Vertical femoral neck fractures accompanied by a medial calcar defects are difficult to manage. Stable fixation and functional stability restoration of medial calcar defects are difficult to achieve due to their compromising effects on the structural integrity of the proximal femur. As ensuring sufficient load transmission and rotational stability of the femoral head is crucial, addressing the calcar defect becomes a crucial aspect of the treatment protocol [2].

The optimal management strategies for vertical femoral neck fractures with medial calcar defects have been a topic of continuous deliberation among orthopedic surgeons [3]. Attaining stable fixation while simultaneously addressing the medial calcar defect represents a significant challenge in the management of these fractures. The medial calcar, an indispensable anatomical component responsible for maintaining the structural integrity of the proximal femur, plays a pivotal role in facilitating load transmission and rotational stability. Inadequate management of the calcar defect may jeopardize fracture stability and increase both the likelihood of fixation failure and the incidence of postoperative complications [4]. Several treatment modalities have been suggested for addressing these fractures, such as cannulated screws (CSs), dynamic hip screws, plate osteosynthesis, intramedullary nails (IMNs), hemiarthroplasty, and total hip arthroplasty. Most of femoral neck fractures are common in the elderly and are mostly treated with hemiarthroplasty or total hip arthroplasty [5]. Nevertheless, because there is no comprehensive clinical evidence supporting any specific treatment strategy for these fractures with such an intricate nature, there is no consensus on their optimal treatment strategy, especially in young patients [6].

In addition, when choosing the most appropriate treatment approach for vertical femoral neck fractures with medial calcar defects, several factors need to be taken into account, including patient age, bone quality, degree of fracture displacement, the presence of associated injuries, and the surgical expertise of the orthopedic surgeon [7]. Each treatment option presents its own set of advantages and disadvantages, which further contributes to the absence of consensus among orthopedic surgeons. This situation emphasizes the need for more comprehensive knowledge and evidence-based guidance in managing these challenging fractures [2].

Hence, the objective of this study is to compare three fixation methods (CS, a combination of CS and buttress plate, and IMN) for medial calcar-defected vertical collum femoris fractures through a biomechanical analysis. The mechanical stability and load-bearing capacity offered by each fixation technique were assessed, so the aim of this study was to better understand vertical femoral neck fractures accompanied by medial calcar defects to facilitate the formulation of evidence-based recommendations for their management. It was hypothesized that the application of CS and medial buttress plates in the described fracture types would yield superior biomechanical outcomes.

## Methods

### Specimen preparation

**Fig. 1:**
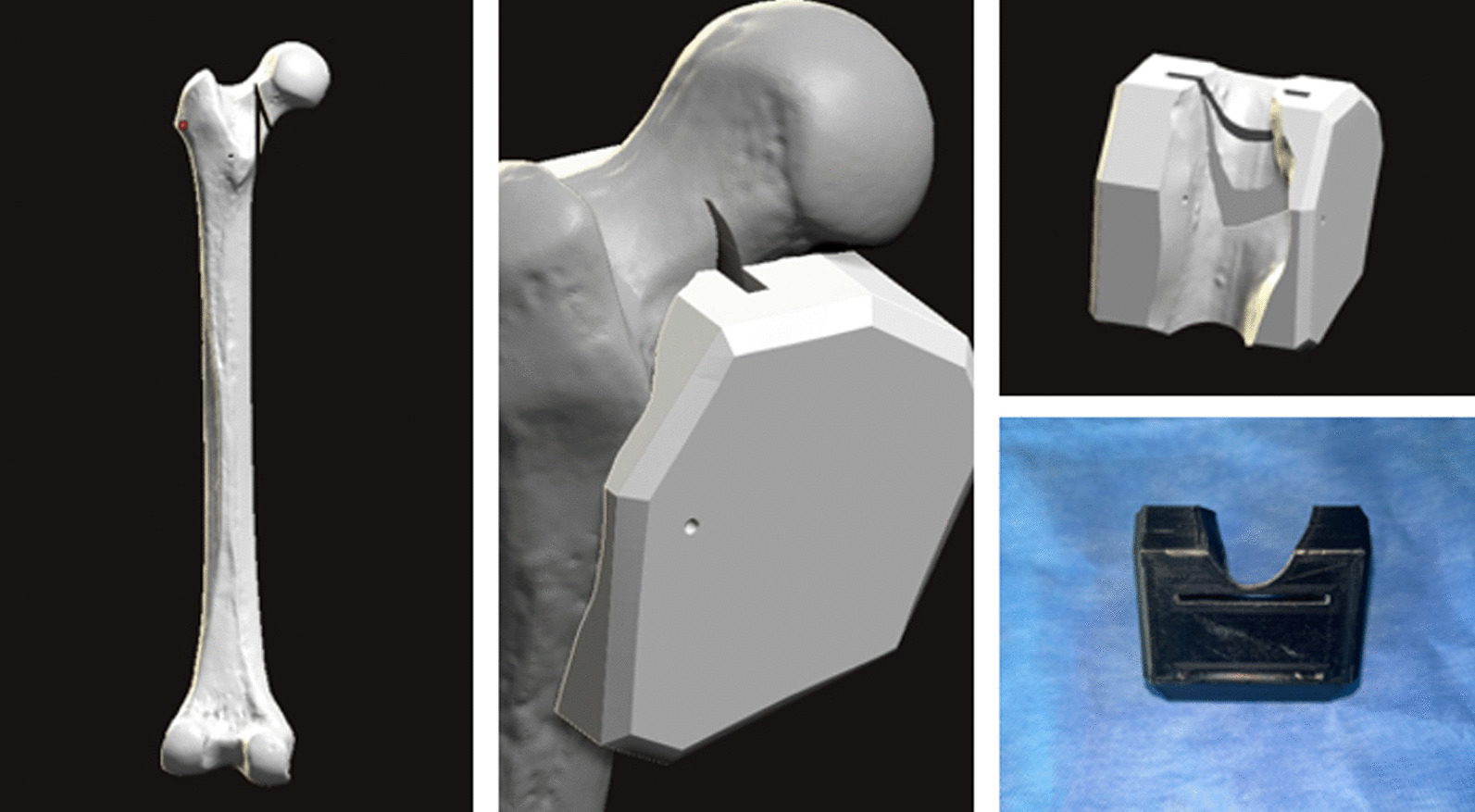
Custom-made saw guide produced to create a homogeneous osteotomy line in all specimens

**Fig. 2:**
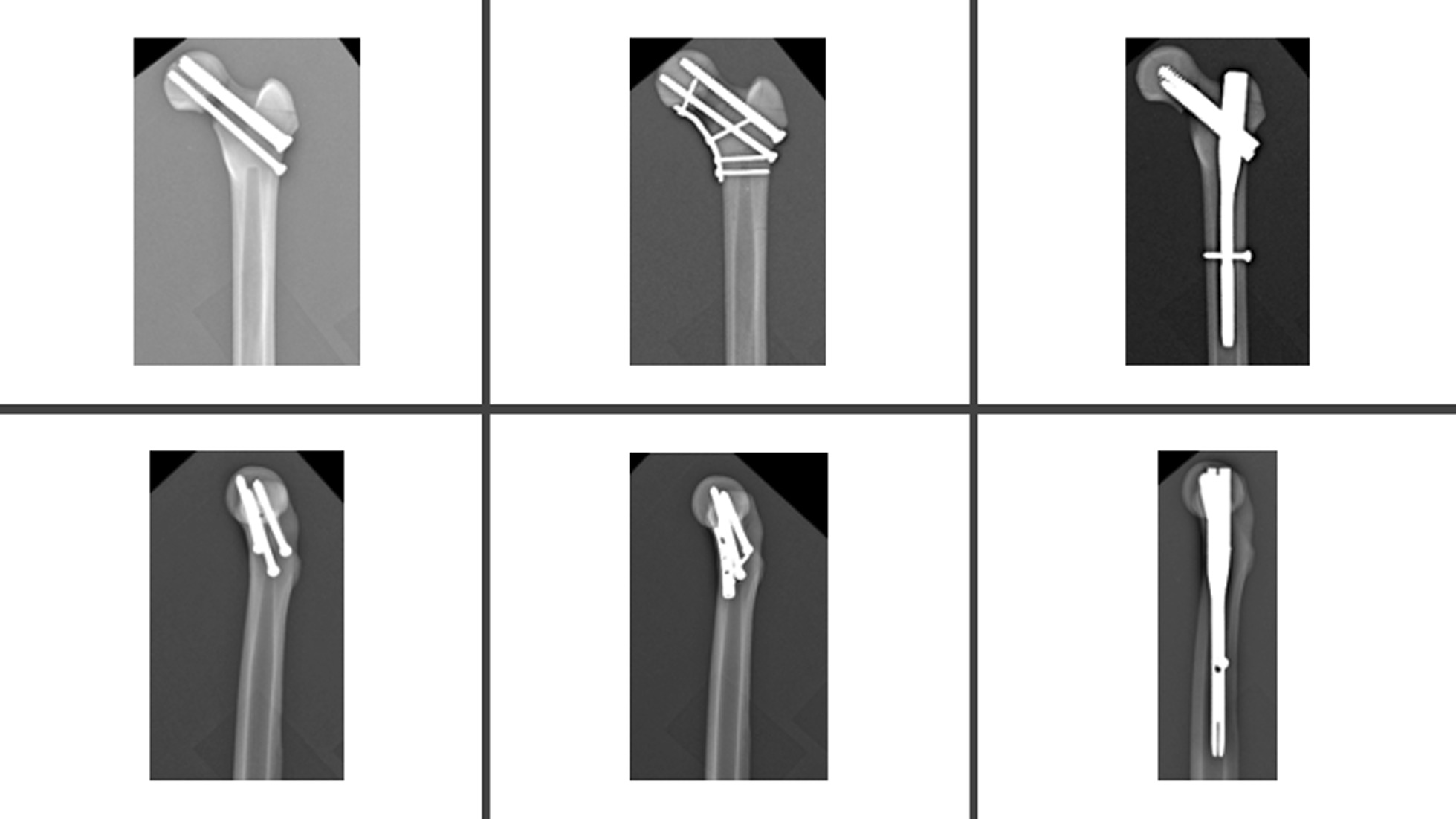
Fluoroscopy-guided fixation was initially applied to ensure proper positioning and continuity of reduction in all specimens. Then, implants were removed to create the osteotomy line, and the specimens were refixed. Following osteotomy and fixation, X-ray was performed on all specimens

In the study, synthetic composite bones were subjected to tests to ascertain their load-bearing capacity until failure and assess their stiffness properties. Using data obtained from the pilot study, a power analysis was performed, which revealed that a minimum of 5 models per group was required to detect a statistically significant difference of 10% between the groups, taking into account a calculated standard deviation of 5% [8, 9]. For the experimental procedure, a standard synthetic model of the proximal femur bone (Synbone no: 2250.01, Length: 465 mm, Condylar width: 86 mm, Neck angle: 135°, Anteversion: 15°, Head diameter: 48 mm, Canal diameter: 10 mm, Graubünden, Switzerland) was used, and each group consisted of seven specimens. To ensure consistent results, a single surgeon performed the surgical procedure on all specimens. The distal femoral condyles were removed by cutting approximately 30 cm below the tip of the greater trochanter using a saw. To ensure uniform fracture configurations in all synthetic bone models, the models underwent scanning using a 3D scanner (Einscan Shining HX, SHINING 3D, Hangzhou, China). Subsequently, a 3D-printed bone-specific cutting guide (Raise 3D Premium ABS, Ultimaker S5 Raise Pro2 3D, Utrecht, the Netherlands) was created based on the vertical femoral neck fracture with a medial calcar defect (Pauwels type 3) (Fig. 1). Using the custom-made saw guide, standardized fractures were meticulously created. To ensure accurate and anatomical reduction and fixation, the specimens were first fixed using three different methods and then osteotomized. All of these procedures were performed under fluoroscopic guidance. In Group 1, three CSs were utilized for fixation, while in Group 2, a combination of three CSs and a medial buttress plate was used. Group 3, on the other hand, received an IMN. The diameter and length of each implant were carefully assessed, and osteotomy and osteosynthesis were performed under fluoroscopic guidance. Additionally, two-directional X-ray imaging was conducted following osteotomy and fixation (Fig. 2).

### Surgical technique

In Group 1, three 6.5-mm cannulated screws (100 mm, 100 mm, 95 mm; Ortip, Istanbul, Turkey) were inserted in an inverted triangular pattern into the femoral head. The initial screw was positioned close to the calcar and inserted into the subchondral bone of the femoral head. The second and third screws were inserted in an anterosuperior and posterosuperior direction, respectively, maintaining parallel alignment with the first screw. In Group 2, fracture fixation was achieved using a combination of a three-cannulated screw and a medial buttress plate (5-hole 1/3 tubular plate, two 40 mm screws, one each 36 mm screw and 28 mm screw; Ortip, Istanbul, Turkey) placed in the defect area. In Group 3, after reaming the medullary cavity, an IMN (10 × 150 mm; Smith & Nephew, Memphis, USA) was inserted with the assistance of an aiming device. Subsequently, the lag screw was positioned in the middle of the femoral neck. Radiologically, it was ensured that the lag screw placement had a tip-apex distance < 25 mm [10]. Using the lag screw as a guide, the compression screw was carefully inserted, ensuring continuous rotational stability until achieving anatomical alignment. Finally, the nail was statically locked in place. All surgical procedures were performed with fluoroscopic guidance.

### Biomechanical testing

**Fig. 3:**
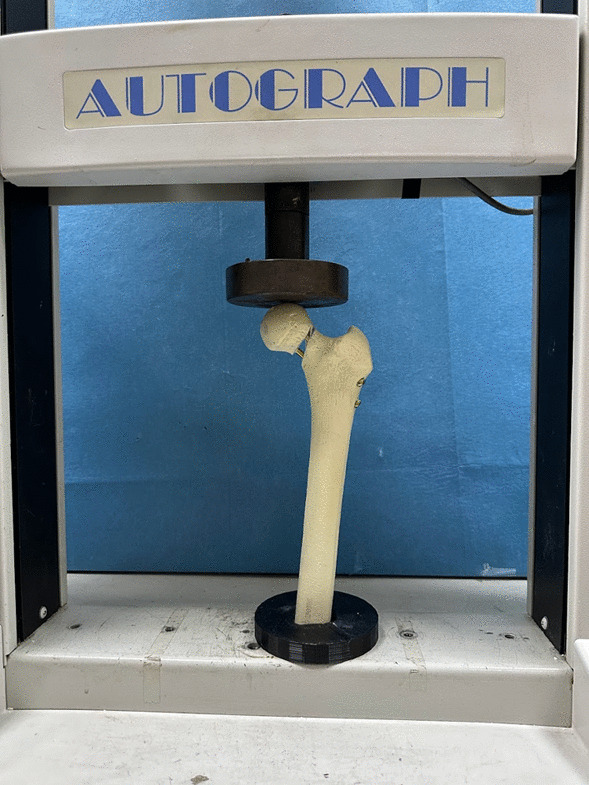
Biomechanical test setup with femur specimen instrumentation with cannulated screws

The vertical loads were applied to the femoral heads at 16° to the midline using a servohydraulic universal testing machine (Shimadzu Autograph AGS, Kyoto, Japan) (Fig. 3). To prevent artificial dislocation and mimic the minimal physiological load of the hip joint during the swinging phase, a constant preload of 100 Newton (N) was maintained throughout the testing series [11]. The failure load and displacement values were determined under axial loading conditions in a simple ramp from preload to failure with a loading velocity of 10 mm/minute. Stiffness values were calculated by analyzing the linear portions of the load–displacement curves obtained in units of N/mm. Failure was determined by the occurrence of any of the following: fracture in the femoral neck or shaft, cut-out/cut-through, implant failure, or a sudden decrease in load resistance observed on the load–displacement curve. The load level at which failure occurred was identified as the ultimate failure load.

### Statistical analysis

Normality was tested using the Shapiro–Wilk test. One-way ANOVA and Tukey’s HSD post hoc test were used for comparisons. Statistical evaluations were performed using the SPSS software package (SPSS, Chicago, IL). The level of significance was set at *p* < 0.05.

## Results

**Fig. 4:**
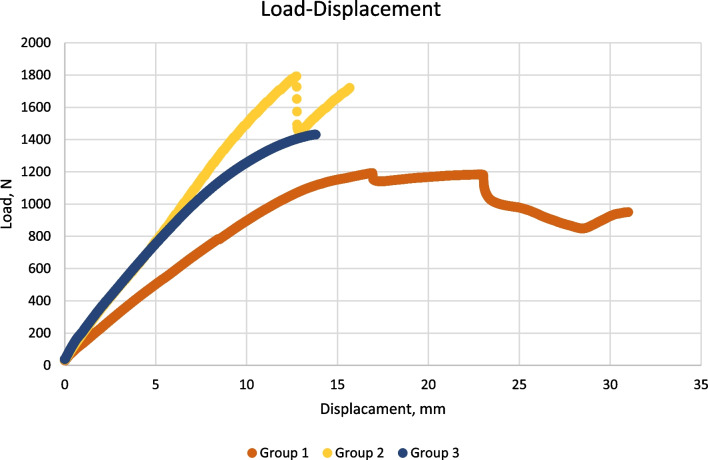
Load–displacement curve of one sample from each group

**Fig. 5:**
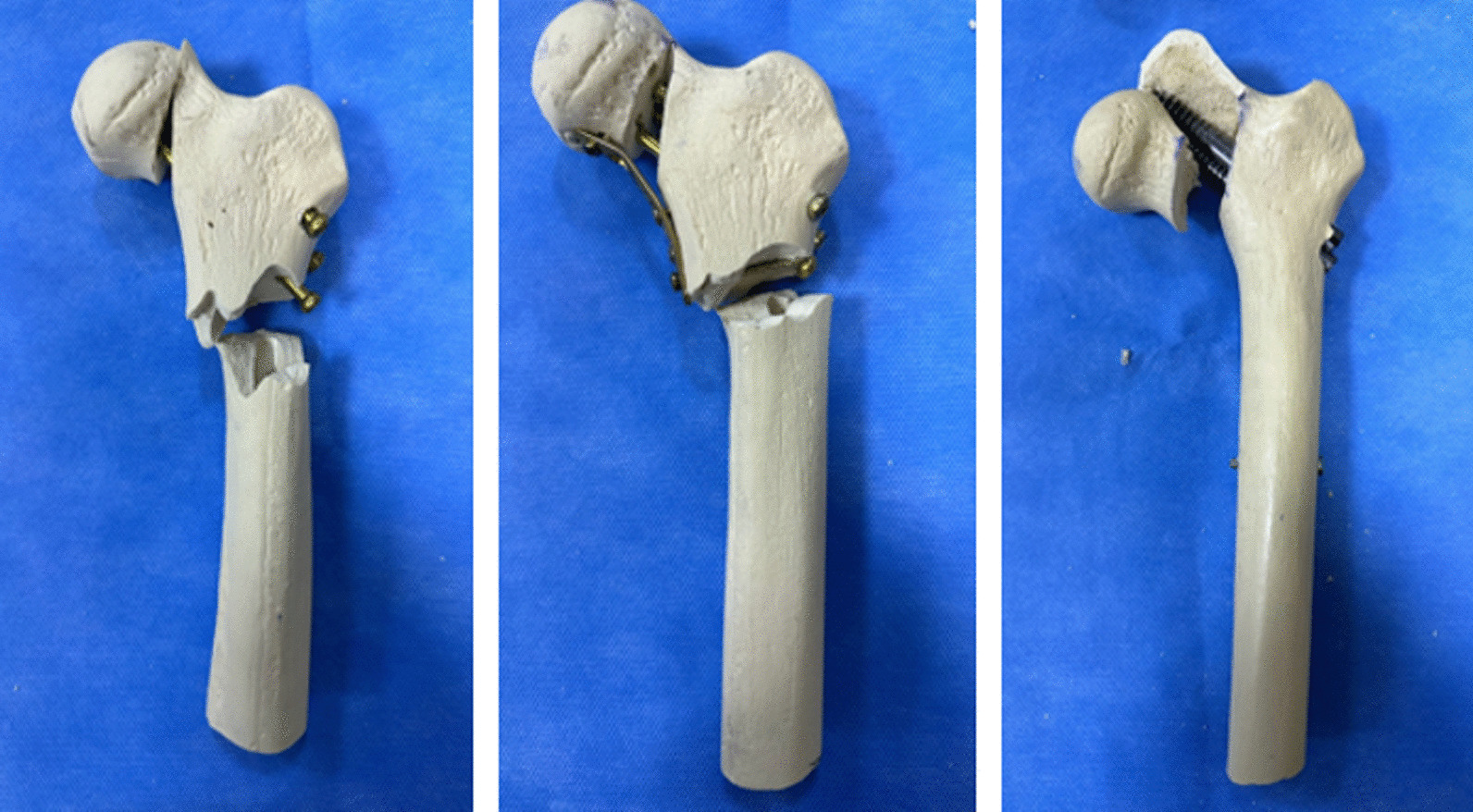
The characteristics developed after failure of one sample from each group.

In the statistical analyses, the load to failure values were determined as follows: Group 2 > Group 3 > Group 1 (Fig. 4). A significant difference was found in the comparison of the load to failure values among the groups (Group 1 vs. 2, Group 1 vs. 3, Group 2 vs. 3 *p* < *0.05*). The load to failure is the load carried by the specimen at the moment when one of the failure conditions described above occurs. When comparing the stiffness values, it was determined that Group 2 had the highest mean, while Group 3 had the lowest mean. In the comparison between groups, the difference between Group 2 and Group 1 was found to be statistically significant. The average displacement amounts of the specimens under the maximum load were observed as follows: Group 1 > Group 3 > Group 2. However, the comparison between the groups in terms of displacement values did not reveal a statistically significant difference (Table 1). The fracture and displacement patterns observed at the point of failure load in the specimens of the groups under axial loading are as follows: In all specimens of Group 1, the displacement occurred at the existing fracture line, and a new fracture occurred at the subtrochanteric region. All specimens in Group 2 only fractured at the subtrochanteric region. The specimens in Group 3 were displaced from the existing fracture line, and the loading test was terminated. In 2 specimens in Group 3, the fracture occurred at the level of the distal static locking screw, and the loading test was terminated (Fig. 5).

**Table 1:** Statistical analysis of the biomechanical properties of the groups

	Group 1 (mean ± SD)	Group 2 (mean ± SD)	Group 3 (mean ± SD)	*p*
Load to failure (Newton)	1008.57 ± 185.75	1794.09 ± 195.87	1435.39 ± 101.27	Group 1 vs. 2 *p* < 0.05*
				Group 1 vs. 3 *p* < 0.05*
				Group 2 vs. 3 *p* < 0.05*
Stiffness (Newton/mm)	89.7 ± 9.71	175.08 ± 48.03	134.67 ± 31.56	Group 1 vs. 2 *p* < 0.05*
				Group 1 vs. 3 *p* = 0.06
				Group 2 vs. 3 *p* = 0.09
Displacement (mm)	17.19 ± 5.37	13.17 ± 1.92	14.28 ± 4.73	Group 1 vs. 2 *p* = 0.21
				Group 1 vs. 3 *p* = 0.43
				Group 2 vs. 3 *p* = 0.88

SD: Standard deviation *p < 0,05

## Discussion

The primary finding of the study is that fixation with a medial buttress plate in addition to cannulated screws in vertical, unstable fractures with a medial calcar defect provides an additional biomechanical contribution. Furthermore, it has been observed that intramedullary nail fixation of the described fracture types is more durable than fixation with cannulated screws alone. However, when evaluating implant options, considering only the maximum load they can bear is not an appropriate perspective. The susceptibility to deformation and resistance of the implants are also important parameters. In this evaluation, the concept of stiffness is used [12]. In the comparison of stiffness, it was statistically confirmed that the cannulated screw and medial buttress plate achieved significantly higher results than other implant options. In light of these findings, it can be concluded that Group 2 better managed vertical, medial defected fractures.

Fractures occurring at the hip joint typically occur in the femoral neck area, and the probability of a fracture in the trochanteric region increases with age [13]. Hence, in younger adults, hip fractures are significantly more likely to occur in the neck region than in other regions of the hip. According to the Pauwels classification, unstable vertical fractures (type 3) are common in young adults and necessitate precise reduction and secure internal fixation [14]. Despite the availability of a wide range of techniques for the fixation of femoral neck fractures, the incidence of complications remains unacceptably high, and there is a lack of consensus regarding the most appropriate technique to treat these fractures. The objective of femoral neck fracture fixation is to establish adequate mechanical stability until the fracture has fully healed. Especially for comminuted and defected fractures, the stability of the osteosynthesis construct depends greatly on the mechanical stability of the implant [7]. In the treatment of femoral neck fractures, cannulated screw systems are one of the methods employed. The stability of this method depends on various factors, such as screw type, number of screws, screw thickness, position, orientation, and configuration. In biomechanical studies, it has been demonstrated that the use of three cannulated screws with a diameter greater than 6 mm in a reverse triangle configuration provides appropriate stability in femoral neck fractures [15–17].

Intramedullary nails have the ability to transfer the loads encountered in the femoral neck to the shaft of the femur. It has been shown that IMNs provide sufficient stability, especially for unstable basicervical fractures and Pauwels type 3 fractures. Biomechanical studies comparing implants for femoral neck fracture fixation in young adults have focused not only on the type of implant but also on the type of fracture, which significantly affects the mechanical performance [18–21]. The use of CS systems in vertical, unstable fractures has been observed to result in earlier varus collapse [22]. IMNs have been used in the described unstable fracture types and have provided better mechanical stability than CS systems [23]. The results of this study also demonstrated, in line with the literature, that the group treated with IMNs could resist higher loads than the group treated with CS.

Despite all this information, there is still no definite consensus on whether inverted triangle configuration CS fixation or IMN fixation is sufficient to prevent complications such as nonunion or refracture in unstable femoral neck fractures [24]. Despite being the most commonly used method due to its torsional stability and minimal disruption of femoral head blood supply in intracapsular femoral neck fractures, the use of CSs is still a concern in young adults with vertical, unstable fractures [25]. While it has been reported in the literature that unstable femoral neck fractures have been successfully treated with proximal femoral nails with anti-rotation properties, there are only a few studies available that have reported positive outcomes of IMN usage for such fractures [24, 26]. Although there is limited research available, IMNs should be considered an option for the fixation of these fractures due to their favorable biomechanical properties, such as having a short lever arm on the implant and reduced bending moment, as well as being minimally invasive [24]. Additionally, IMNs can transfer bending moments from the femoral head and neck region to the cortical bone of the femoral shaft, and CSs can transfer moments only between the screws and cancellous bone [27]. Furthermore, in another study, it has been reported that minor inaccuracies in the placement of IMN and screws did not significantly affect the success of the entire fixation in stable proximal femur fractures. These findings underscore IMN advantageous in terms of ease of application as well as biomechanical benefits [28].

It is well known that maintaining the stability of reduction and fixation is the key to fracture healing. With this goal in mind, the use of medial buttress plates and cannulated screws in an inverted triangle configuration has recently become a popular method. This technique provides sufficient stability in unstable and comminuted fractures. In the literature, the usage of additional medial buttress plates has been reported to increase stability against vertical shearing forces [4, 25]. Furthermore, in a study, it was hypothesized that a plate implanted on the medial vertex in vertical, unstable fractures could function as a buttress, resist shear forces and transform them into compression forces. Based on this, it has been claimed that plate characteristics may reduce the rates of complications associated with reduction loss, which are commonly observed in these types of fractures [4]. In another study, it was stated that the addition of medial plate implantation to traditional CS fixation provides biomechanical advantages, leading to increased rates of union [29]. However, the procedure requires the opening of the joint capsule and may have a negative impact on the blood supply of the femoral head. Although the authors state that plate fixation does not disrupt the primary blood supply to the femoral head, the application of a medial buttress plate has not yet become popular due to the complexity of the surgical technique, thus further clinical studies are needed [24]. Additionally, safe points have been identified to ensure that medial calcar plate fixation does not disrupt the blood supply to the femoral head. Accordingly, it has been stated that the 6 o'clock position is the safest zone for application [30, 31]. Considering the biomechanical contributions of medial buttress plate implantation, its ability to preserve femoral head vascularity, and its lack of disadvantages aside from its challenging procedure, it can be concluded that medial buttress plating may be beneficial for young patients with unstable and defective fractures [25, 32].

Considering that the load generated by daily activities around the hip is approximately 1400–1500 N, it was observed in this study that IMNs and medial buttress plate implantation with CSs for the fixation of the simulated fracture type were able to resist these loads, while the average value of the CS-only group remained below these values [33]. It is necessary to evaluate the data not only biomechanically but also functionally and physiologically. In studies conducted, it has been found that young adults with unstable vertical fractures who received additional plate application with cannulated screws had higher hip functional scores and higher fracture healing rates than patients who received cannulated screws only [29, 34]. In another study evaluating the biological, social, and functional outcomes of patients who underwent IMN and plate fixation, it was claimed that IMNs caused less blood loss than plate fixation, had a shorter recovery period, shorter hospital stay, and better functional outcomes [35]. Therefore, when considering suitable implant options for the treatment of challenging fractures, it is important to not solely rely on biomechanical characteristics.

This study has several limitations. First, it is probable that there are additional forces exerted on the proximal femur during the occurrence of these fractures, which were not included in our model. Furthermore, this study did not simulate soft tissues such as muscles, the joint capsule and ligaments, which are crucial for hip stabilization and function. Another limitation is the loading test of this study. The femora were subjected to failure rather than cycling them at a lower force until failure. One significant limitation of this study is the omission of torsional stability testing. However, it is important to note that axial load is considered to be the primary deforming force responsible for fixation failure in hip fractures. Additionally, the use of synthetic bones was another limitation in this study. However, it was a controlled variable, as all experiments were conducted using identical models from the same batch. Finally, dynamic hip screws, which are known to be used in the treatment of vertical, comminuted, unstable fractures, were not used, which can also be considered a limitation.

## Conclusion

This study has demonstrated that medial buttress plate application in the fixation of vertical, unstable, medial calcar-defected femoral neck fractures significantly enhances fracture stability and improves resistance to deforming forces. Additionally, in these fractures, IMN fixation is comparable to other fixation options.

## Data Availability

The datasets used and/or analyzed during the current study are available from the corresponding author on reasonable request.

## Abbreviations

CS: Cannulated screw
IMN: Intramedullary nail
N: Newton
